# Phenotypic and genotypic correlation between myopia and intelligence

**DOI:** 10.1038/srep45977

**Published:** 2017-04-06

**Authors:** Katie M. Williams, Pirro G. Hysi, Ekaterina Yonova-Doing, Omar A. Mahroo, Harold Snieder, Christopher J. Hammond

**Affiliations:** 1 Department of Ophthalmology, King’s College London, 3rd Floor Block D South Wing, St Thomas’ Hospital, Westminster Bridge Road, London, SE1 7EH, UK; 2 Department of Twin Research & Genetic Epidemiology, King’s College London, 3rd Floor Block D South Wing, St Thomas’ Hospital, Westminster Bridge Road, London, SE1 7EH, UK; 3 Unit of Genetic Epidemiology & Bioinformatics, Department of Epidemiology, University Medical Center Groningen, University of Groningen, Hanzeplein 1, 9713 GZ, The Netherlands

## Abstract

Myopia, or near-sightedness, is our most common eye condition and the prevalence is increasing globally. Visual impairment will occur if uncorrected, whilst high myopia causes sight-threatening complications. Myopia is associated with higher intelligence. As both are heritable, we set out to examine whether there is a genetic correlation between myopia and intelligence in over 1,500 subjects (aged 14–18 years) from a twin birth cohort. The phenotypic correlation between refractive error and intelligence was −0.116 (p < 0.01) - the inverse correlation due to the fact that myopia is a negative refractive error. Bivariate twin modeling confirmed both traits were heritable (refractive error 85%, intelligence 47%) and the genetic correlation was −0.143 (95% CI −0.013 to −0.273). Of the small phenotypic correlation the majority (78%) was explained by genetic factors. Polygenic risk scores were constructed based on common genetic variants identified in previous genome-wide association studies of refractive error and intelligence. Genetic variants for intelligence and refractive error explain some of the reciprocal variance, suggesting genetic pleiotropy; in the best-fit model the polygenic score for intelligence explained 0.99% (p = 0.008) of refractive error variance. These novel findings indicate shared genetic factors contribute significantly to the covariance between myopia and intelligence.

Myopia, or near-sightedness, occurs when a viewed object is focused in front of the retina resulting in the observer seeing a blurred image. This generally occurs a result of axial elongation, or lengthening, of the eyeball during childhood. Refractive correction in the form of glasses, contact lenses or refractive surgery is required in order for a clear image to be obtained. It is the most common ocular condition and the prevalence is increasing globally, most dramatically in urban East Asia^1^,^2^,^3^,^4^.

Environmental factors are known to play a key role in myopia risk. There are well-established links between myopia and urbanization, lack of time outdoors, reduced light exposure, socio-economic status, prenatal factors, near work, and educational attainment^5^,^6^,^7^,^8^,^9^,^10^,^11^,^12^. The latter is particularly well replicated in populations around the world^3^,^13^,^14^,^15^ and may reflect a number of predisposing factors; less time outdoors whilst studying, more time on near work activities, or higher intelligence. Associations between higher intelligence quotients (IQ) and myopia have been reported since Cohn’s first observation in 1883^16^, and subsequently in various studies internationally^15^,^17^,^18^,^19^,^20^,^21^,^22^,^23^,^24^. The relationship appears consistent in children and adults, across a range of IQ tests, and is independent of years of education completed^15^,^25^. Recent prospective, pediatric studies have reported up to twice the risk of myopia for those in the highest IQ quartile^18^,^26^.

However, the nature of the association between myopia and IQ remains poorly understood. While it may reflect inadequate adjustment for reading/time outdoors, one potential explanation is that myopic children, with their cumbersome glasses, may be less likely to play sports outdoors and more likely to spend time on their school studies^27^, thus attaining their full ‘potential’ in educational and IQ tests. Conversely, more intelligent children may spend more time reading and studying with less time in the protective outdoor environment, thereby increasing their chance of developing myopia. An alternative, but not mutually exclusive, hypothesis is that myopia and IQ may share a pleiotropic relationship^28^,^29^. Pleiotropy implies that a single gene may influence one or many apparently unrelated phenotypes, but to date this theory has not been tested in respect to myopia and IQ. Shared genetic factors pre-determining the risk for both traits is an interesting hypothesis made more plausible by the fact that both traits are significantly heritable; refractive error is 70–80% heritable^30^,^31^, whilst IQ is 30–60% heritable, increasing with age^32^. Recent genome-wide association studies (GWAS) have identified genetic variants associated with refractive error and with childhood IQ^33^,^34^.

The twin model provides the ‘perfect’ natural experiment to examine the relative effect of genes and environment on trait variation. The technique allows global estimation of genetic and environmental effects regardless of underlying genes or specific environmental factors^35^. Previous twin research has suggested shared genetic factors between refractive error and axial length, and, albeit with limited power, myopia and educational attainment^36^,^37^. In this study we explore to what extent the genetic risk between IQ and myopia is shared, utilizing a longitudinal twin birth cohort, the Twins Early Development Study (TEDS). Additionally, using genome-wide genotyping data, we created polygenic risk scores from genome-wide association studies of myopia and of IQ to predict the variance of the alternate trait.

## Results

Data on refractive error and IQ were available for 1529 twin subjects, summarized in Table 1. The prevalence of myopia (≤−0.75 Dioptres (D)) in the sample was 26.7%. Individuals with myopia were significantly older (17.0 vs 16.1 years, p < 0.01) and had a greater proportion of non-white ethnicity (93.8% vs 96.5% white ethnicity, p < 0.01). The academic achievement (93.0 vs 87.9 mean total General Certificate of Secondary Education score, p < 0.01), IQ scores (0.24 vs 0.02 general cognitive ability ‘g’ score, p < 0.01), and maternal educational levels (4.64 vs 4.16 mean highest educational level, p < 0.01) were all significantly higher in those with myopia.

**Figure 1:**
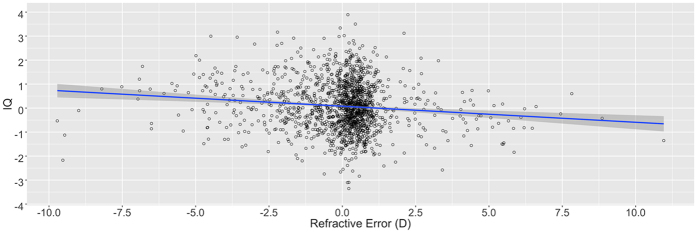
Scatter plot of refrective error against IQ (both variables adjusted for age and sex) with linear regression line and 95% confidence region [n = 1529].

The phenotypic correlation between refractive error and IQ, adjusted for the effects of age and sex, was −0.116 (p < 0.01) [Fig. 1] - the negative correlation is due to the fact that myopia constitutes a negative refractive error. In a univariant linear model IQ was significantly associated with refractive error (beta coefficient (β) −0.217 p < 0.01, adjusted for relatedness only) and explained 1.5% of the variance. In a multiple linear regression model incorporating known associations with myopia (namely age, sex, ethnicity, maternal education, academic achievement and time spent reading books) IQ remained a significant predictor (β −0.145, p = 0.02). Logistic models (adjusted for significant associations in univariant analyses) revealed an odds ratio (OR) for myopia of 1.18 with increasing IQ scores (p < 0.01, 95% Confidence Interval (CI) 1.02–1.37), and for those in the highest IQ quartile the risk of myopia was one and half times greater compared to those in the lowest quartile (OR 1.56, p = 0.02, 95% CI 1.08–2.25).

### Twin modeling

**Figure 2:**
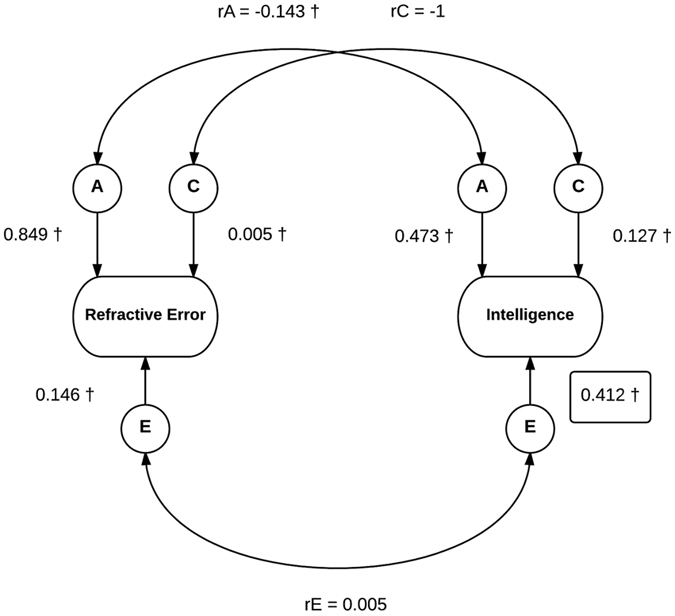
Bivariate twin ACE model for refractive error and IQ. Path estimates with 95% confidence intervals; A = additive genetic factors, C = common environmental factors, E = unique environmental factors; ^†^=significant path estimates [n = 1529].

Bivariate twin modeling estimated the heritability of refractive error at 85% (95% CI 79.9–87.5) and at 47% (95% CI 36.7–57.8) for IQ [Fig. 2]. Shared environmental factors contributed significantly to IQ variance (13%, 95% CI 3.9–21.2) and a lesser extent to refractive error (0.5%, 95% CI < 0.01–5.0). Individual environmental factors accounted for 15% (95% CI 12.3–17.4) of refractive error variance and a greater proportion of IQ variance (40%, 95% CI 36.5–43.9).

The genetic correlation (*rA*), that is the correlation between the genetic influences on refractive error and the genetic influences on intelligence, was −0.143 (95% CI −0.013 to −0.273). Shared genetic effects can be estimated as follows: *√A(Refractive Error) x rA x √A(IQ*). This gives an estimate of 0.091. Therefore, it can be calculated that the proportion of phenotypic correlation between refractive error and IQ due to shared genetic effects is 78%.

### Polygenic Risk Scores

**Figure 3:**
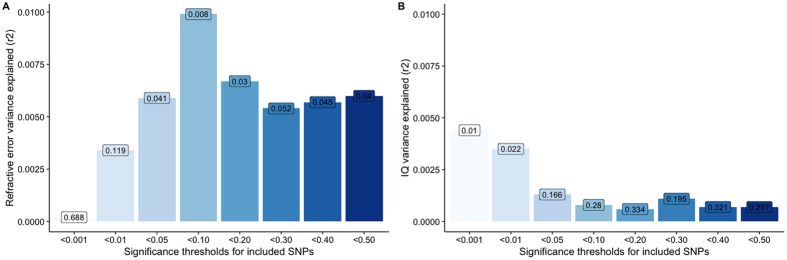
Genome-wide polygenic scores for intelligence (**A**) and refractive error (**B**) respectively predict variance in refractive error and intelligence. Polygenic risk scores were created using different significance thresholds for inclusion of SNPs (<0.001 to <0.50). The uncorrected p-values above each bar indicate the statistical significance of the association between the polygenic score and the respective trait. (**A**) n = 696, adjusted for two principal components, age and sex. (**B**) n = 1517, adjusted for two principal components, age and sex.

Polygenic risk scores (PRS) can be used to estimate the degree of phenotypic variance explained by the contribution of thousands of common genetic variants (SNPs) previously associated with the trait of interest or, in this analysis, an alternate trait. PRS for childhood IQ were calculated for all unrelated individuals in TEDS with genome-wide association data using eight thresholds of significance for inclusion of variants (see Methods) from the results of a GWAS for child IQ, excluding TEDS^33^. In TEDS the IQ PRS explained 1–3% of the variance of IQ (results not shown). In all PRS models, a higher IQ PRS was associated with lower refractive error (i.e. myopia). Refractive error variance predicted by the differing PRS threshold models in 696 individuals are illustrated in Fig. 3(A); refractive error variance explained was approximately 0.5–1% across all models. In the best-fitting model, a PRS (consisting of 19,318 SNPs associated with IQ at the <0.1 p-value threshold) explained 0.99% of the variance (uncorrected p = 0.008). For comparison 3.4% of refractive error variance was explained by SNPs directly associated with refractive error in a GWAS of adults^34^.

Similarly, a polygenic risk score for refractive error was calculated on all genotyped participants using eight thresholds of significance from the results of a GWAS for refractive error in adults^34^. In TEDS the refractive error PRS explained 1–2.5% of refractive error variation (results not shown), comparative to the aforementioned figure of 3.4% variance explained in adults^34^. Again, a lower refractive error PRS (ie. myopia) was associated with a higher IQ. The results of the different PRS threshold models in 1517 individuals are illustrated in Fig. 3(B); IQ variance explained was approximately 0.1–0.4% across the models. In the best-fitting model, 178 refractive error-associated SNPs explained 0.44% of IQ variance (uncorrected p = 0.01).

## Discussion

Twin participants with high IQ (highest quartile) were one and a half times more likely to be myopic, comparable to others^18^,^26^. IQ alone explained 1.5% of refractive error variance and remained a significant predictor when adjusted for educational attainment, contrary to others^38^. In twin modeling both traits were heritable (refractive error 85%, IQ 47%), and genetic factors explained the majority (78%) of the phenotypic correlation (r = 0.12) between IQ and refractive error. Reciprocal genome-wide PRS significantly predicted the variance of both refractive error (~1%) and IQ (~0.4%). These analyses provide evidence for genetic correlation between myopia and IQ, with shared genetic factors underlying a small proportion of variance in both traits.

To our knowledge no previous study has examined the extent of shared genetic factors for myopia and IQ. Previous bivariate twin analyses for myopia have examined the relationship with axial length and with educational attainment^36^,^37^. Dirani *et al*. reported that 76% of the phenotypic sharing between refractive error and educational attainment was due to shared genetic factors^36^, similar to the findings in this study, however their genetic correlation was not statistically significant. Genome-wide PRS for IQ and refractive error significantly predict variation in refractive error and IQ respectively. The reciprocal PRS identified genetic effects on both traits, thus indicating a degree of genetic pleiotropy without the implication of any causal direction of effect. The degree of trait variance explained was greatest for the IQ PRS and refractive error (0.99%). Interestingly only 3.4% of refractive error variance in adults is explained by genetic variants specifically associated with the refractive error in previous GWAS^34^, and in our analysis this figure was 1–2.5%, which is not incomparable to the variance explained by ‘IQ SNPs’.

The argument that highly intelligent children, who may spend more time on near-work activities, increase their risk of developing myopia has long been favored^8^,^9^,^39^. However the association between near-work and myopia is inconsistent^40^,^41^, and importantly refraction in young children, prior to the experience of intensive education and near-work, can significantly predict those who will later become myopic^42^,^43^. The possibility that increased time studying can increase an individual’s IQ has been largely discounted; there is no robust evidence that a large effect on IQ can be achieved by such an intervention^44^. Shared genetic factors may play a role in both traits. Early proponents for this theory identified an association between myopia and IQ where the intellectual gain preceded the development of myopia, and differential status in siblings^28^,^45^,^46^. A single myopia gene that influenced brain development with evolutionary advantages for urbanized living was proposed^28^,^29^,^47^. The idea of a single gene controlling brain development and eye growth, in light of modern knowledge of the polygenicity of both traits, now appears implausible. However, the possibility of a number of genes of small effect, perhaps inherited simultaneously and linked, that control neural signaling influencing ocular growth and learning abilities remains interesting. Recent research has identified gene-environment interactions between educational attainment and myopia^48^, whilst PRS for educational attainment predict refractive error and when incorporated as an instrumental variable in Mendelian randomization (MR) analysis support the notion that educational attainment is causally related to refractive error^49^. The education PRS in the MR study only explained 0.25% of refractive error variance, comparatively less than the variance explained by IQ in our analysis. We would argue that IQ may be a ‘purer’ phenotype for analysis than educational attainment, which incorporates many underlying factors.

In axial myopia, the commonest form of myopia, elongation of the eye results in a focused image falling in front of the retinal plane. The retina, together with a number of other ocular structures, including the ciliary body which controls accommodation (near focus), originate embryonically from the same tissue as the brain (neuroectoderm)^50^. Retinal signaling, through detection of focused light, influences scleral remodeling and ultimately ocular axial length^51^,^52^. Therefore, could the same genetic factors in the retina and brain theoretically be involved in the regulation of both structures? There is some evidence that people with high IQ have a ‘larger brain’, with correlations estimated at 0.38 to 0.45^53^,^54^. Brain size itself does not predict cognitive ability within families^55^, although incorporating other neuroimaging variables can provide a modest prediction of IQ variance^56^.

Globally, myopia is becoming more common and IQ scores are rising^1^,^57^. Some have argued that an evolutionary drive may have increased the human population frequency of pleiotropic genes for higher IQ and myopia^58^. The main limitation of this theory is the temporal relationship; evolutionary changes occur over multiple generations whereas the increases in myopia and IQ scores have been observed within the last century. It is likely for myopia that aspects of modern day childhood are more attributable, but it is interesting to note the heritability of IQ increases over childhood and across the lifespan - this has been attributed to genetic amplification, most likely through gene-environment effects rather than additional genetic influences, as the same genes appear to be involved in cognitive ability at different ages^59^,^60^.

This analysis utilizes a powerful twin data set with phenotypic and molecular genetic data; for the IQ PRS comprising of SNPs over a significance threshold of 0.1, we have enough statistical power to detect a genetic covariance of 0.09 between IQ and refractive error^61^. Although the gold standard of cycloplegic autorefraction was not used, at age 14–18 the subjects were old enough for subjective refraction, using techniques to avoid excessive diagnosis of myopia such as use of the duochrome bar. In large epidemiological studies of adults this method introduces minimal bias^62^, whilst in younger populations it has been found that whilst there is a large degree of inaccuracy in children <10 years, in older teenagers the degree of potential inaccuracy is less^63^. The association with IQ was examined using a composite variable (g); this composite score was comprised of verbal and non-verbal tests created to reliably measure general cognitive ability at each age. The technique of twin modeling provides an upper limit estimate of heritability and therefore genetic relationships, whilst it tends to underestimate the effect of shared environmental factors^31^. Although an ADE twin model was marginally better-fitting than the ACE model for refractive error (providing the same heritability estimate of 86%), we elected to use matched ACE models for both traits as this enabled the examination of any potential differences in shared and unique environmental effects. Polygenic risk scores are limited to testing the effect of common genetic variation and the additive genetic model of inheritance, unlike twin modeling. This means the captured genetic contribution is an underestimate of the genetic contribution to trait variance, which may additionally include rare variants, gene-environment interactions and epigenetic effects. Polygenic risk scores are designed to test whether SNPs that do not reach genome-wide significance in a discovery GWAS explain a significant proportion of variation in a trait in an independent sample. The fairly liberal thresholds used will mean many non-associated SNPs are in the score and for this reason the term ‘polygenic risk score’ rather than ‘genetic risk’ is used. The premise is that collectively these SNPs account for a substantial proportion of variation. Despite appropriate measures for adjusting for ancestry, genetic heterogeneity between TEDS and the discovery GWAS results used may influence the degree of association in polygenic risk scores, although we suspect observed differences in association are more likely to be due to the differing success of the discovery GWAS. Polygenic risk scores were also subject to limited power as the sample of genotyped twins was relatively small. The p values have not been corrected for the issue of multiple testing which is a limitation. GWAS for intelligence on larger sample sizes have been performed^64^,^65^; however, these were conducted on adult participants and explain a smaller amount of IQ variance in TEDS compared to that explained by the childhood IQ GWAS used for this study^33^. Finally, no directionality or causality can be inferred from these methods, and the complex effect of gene-environment interaction is not incorporated.

In summary, our bivariate twin model suggests that shared genetic factors underlie the majority of the phenotypic correlation between myopia and IQ. This was substantiated using molecular genetic data where approximately 1% of refractive error variance was explained by genetic variants linked to IQ. This provides novel evidence for a modest but significant contribution of pleiotropic genetic factors contributing to the development of myopia and higher intelligence.

## Materials and Methods

### Participants

The Twins Early Development study (TEDS) is a longitudinal birth cohort of twins studied from a neurodevelopmental perspective using multivariate quantitative and molecular genetic techniques. In the initial TEDS study over 15,000 families of twins born in England and Wales in 1994, 1995 and 1996 were recruited. The sample remains representative of the UK population^66^. Ethical approval for all experimental protocols TEDS and the TEDS myopia study has been provided by the Institute of Psychiatry ethics committee. All methods were carried out in accordance with relevant guidelines and regulations. A subset of 2625 families was selected for the TEDS Myopia study. This sample was selected to include families from TEDS where twins had completed a questionnaire that included eyesight questions, and additional families where twins had genotype data. We excluded from the analyses children with severe current medical problems and families who were not contactable or who lived overseas.

### Measures

Postal questionnaires were sent to 2625 families inviting participation in the myopia study and consent was requested from the parents, as well from the twins, to contact their optician for a recent refraction. A response rate of 51.7% of potential twin participants was achieved (n = 2715). Responders and non-responders were comparable in terms of ethnicity, gender, zygosity, and parental employment; however there was a slightly higher rate of twin and parental secondary school examinations achievement in the responders. Study questionnaires were sent to the optometrists given by 2,283 twins, from whom informed consent was obtained, requesting a brief ophthalmic and refractive history together with a most recent refraction. Non-cycloplegic subjective refractive error measurements were obtained for 1996 individuals (majority 70% aged 16–18, 92% aged 14–18 at their most recent refraction). Spherical equivalent (SE) was calculated using the standard formula (SE = sphere + (cylinder/2)) and the mean of the two eyes was considered for each individual. Myopia was defined as SE ≤ −0.75 diopters (D). Standardized residuals of mean spherical equivalent adjusted for age and sex were calculated (n = 1991).

Multiple child and parent questionnaires, in addition to teacher questionnaires, web-based testing and home assessments, have been conducted over the twins’ life-course. General cognitive ability or g^67^ was assessed using a combination of parent-administered, phone- and web-based tests at ages 2, 3, 4, 7, 9, 10, 12, 14 and 16 years of age. At each age the twins completed at least two ability tests that enabled assessment of verbal and non-verbal intelligence. For this study the measurement of g factor, which is essentially a measure of IQ, was taken from the oldest ages of testing, as this was the age in closest approximation to the age of refraction. At these ages subjects completed a web-based adaptation of Raven’s Standard and Advanced Matrices, and the Mill-Hill Vocabulary Scale^68^,^69^,^70^. Age and sex adjusted standardized residuals at age 16, imputed with age 14 if missing, were calculated for 1529 individuals. Time spent reading was asked on a child questionnaire at the age of 14, where enjoyment and hours spent per week on various hobbies and activities was assessed.

### Genotyping

Genotyping was performed on 3665 individuals (one twin per pair) on Affymetrix GeneChip 6.0 single nucleotide polymorphisms (SNP) genotyping arrays (Affymetrix, Santa Clara, CA, USA) using standard experimental protocols, as part of the WTCC2 project. Twins excluded from genotyping were children with serious medical or perinatal problems, non-white ethnic origin, and English spoken as a second language at home.” A total of 3152 DNA samples (1446 males and 1706 females) survived quality control for relatedness, hetereozygosity, ancestry and hybridization intensity outliers. Genotypes at untyped markers were imputed with HapMap phase II and III, and WTCCC2 controls using IMPUTE (v2). The quality control criteria used to select imputed SNPs were an information score of ≥0.90 for WTCCC2 controls and ≥0.98 for HapMap imputation. Zygosity was assigned using parental questionnaires of physical similarity; this has shown over 95% accuracy when compared to DNA testing^71^. DNA testing was performed when zygosity was unclear.

### Statistical Analysis

Correlation coefficients between refractive error and IQ, adjusted for age and sex, were calculated. The association and variance explained (r^2^ or coefficient of determination) for refractive error with IQ was observed in univariate and multiple linear regression models, adjusting for some of the known risk factors for myopia with refractive error considered as a continuous trait and myopia as a binary trait (≤−0.75 D). The association between different quartiles of IQ and also non-verbal and verbal IQ were evaluated. In analyses p < 0.05 was considered statistically significant.

#### Bivariate twin Modeling

In twin modeling the phenotypic variance of a trait is partitioned into three factors: additive genetic effects (A), non-additive genetic effects (D) or the shared environment between siblings (C), and the individual-specific environment effects (E). Monozygotic pairs (MZ) have the same genetic and shared environmental effects, whereas in dizygotic twins (DZ) additive genetic effects are 50% correlated, but the shared environmental effects are the same. An ACE model, rather than an ADE model, was chosen based on the twin correlations for the traits and in an effort to examine any potential differences in shared and unique environmental effects on the traits. We performed standard ACE model-fitting analysis using the OpenMx package (http://openmx.psyc.virginia.edu) in R (http://www.R-project.org). Heritability (or h^2^) is provided by the estimate for additive genetic effects. A bivariate Cholesky decomposition model for IQ and refractive error was constructed to assess the phenotypic variance and covariance attributable to genetic and environmental factors. There are two mathematically equivalent solutions to the bivariate model and for this analysis we selected the correlated factors solution which does not assume that the factors underlying the first variable influence the second variable and allows ascertainment of the proportion of phenotypic correlation due to A, C & E. Age and sex adjusted standardized residuals were used for both traits. The degree of phenotypic correlation explained by genetic factors was calculated by dividing the estimate of the shared genetic effects by the phenotypic correlation.

#### Polygenic Risk Scores

Polygenic risk scores (PRS) enable estimation en masse of the genetic contribution of thousands of common variants, generally of small individual effects, on the variation of a trait. In this study, estimation, at a participant level, of the degree of variance of one trait (eg refractive error) explained by the SNPs associated with a second trait (eg. IQ) from an unrelated sample was examined. The results from a large international meta-analysis GWAS for IQ^33^ in children of European ancestry were used to calculate an IQ PRS (results selected that did not include TEDS participants), whilst a similar GWAS for refractive error in adults (limited to those of European ancestry) was used to calculate a refractive error PRS^34^. From the GWAS results the SNP, reference allele, beta coefficient and p value were extracted. For each individual the quality controlled SNPs were pruned for linkage disequilibrium using a clumping approach in Plink v1.9 using a pairwise cut-off of r^2^ ≤ 0.25 within 200 kB window, and a MAF cut-off of >0.03. This resulted in 120481 SNPs. Individualized PRS, for both refractive error and IQ, were calculated with eight thresholds for inclusion of trait-associated SNPs, so that the effect of a range of SNPs on the alternate trait could be examined. The eight significance thresholds tested were: p value < 0.001, <0.01, <0.05, <0.1, <0.2, <0.3, <0.4 and <0.5. General linear models were constructed for IQ-associated SNPs and refractive error with age, sex and the first two ancestry-informative principal components were included as covariates in 696 unrelated individuals (one twin per pair), and then vice versa in 1517 individuals. In each model the significance of the PRS (assessed by an uncorrected p-value, with <0.05 considered significant) and variance explained (assessed by r2) was observed. Analysis was performed using Plink v1.9^72^ and Stata version 13.1 (StataCorp. 2013. Stata Statistical Software: Release 13. College Station, TX: StataCorp LP).

## Additional Information

**How to cite this article**: Williams, K. M. *et al*. Phenotypic and genotypic correlation between myopia and intelligence. *Sci. Rep.* **7**, 45977; doi: 10.1038/srep45977 (2017).

**Publisher's note:** Springer Nature remains neutral with regard to jurisdictional claims in published maps and institutional affiliations.

## Figures and Tables

**Table 1:** Twin participant characteristics by zygosity, myopic status and genotype availability.

	Mean age at refraction measurement (years), SD	Mean age at IQ measurement (years), SD	Sex, % male	Ethnicity, % White European	Academic achievement (mean total GCSE score), SD	Maternal education (mean highest education level), SD	Mean time spent reading (hours), SD	Mean refractive error (D), SD	Mean IQ (g), SD
MZ twins (n = 564)	16.5, 1.80	15.9, 1.04	36.6	94.9	90.5, 23.1	4.17, 1.96	5.09, 6.80	−0.61, 1.79	0.00, 0.95
DZ twins (n = 965)	16.4, 1.73	15.7, 1.27	41.2	96.2	90.2, 24.3	4.50, 2.13	4.55, 6.01	−0.30, 1.77	0.12, 1.05
Combined (n = 1529)	16.4, 1.76	15.8, 1.20	39.5	95.7	90.3, 23.9	4.37, 2.07	4.75, 6.32	−0.41, 1.78	0.07, 1.01
Participants with myopia (n = 409)	17.0, 0.82	15.9, 1.12	39.6	93.8	93.0, 24.2	4.64, 2.04	5.10, 7.50	−2.42, 1.57	0.24, 1.02
Participants without myopia (n = 1120)	16.1, 1.92	15.7, 1.25	42.8	96.5	87.9, 23.8	4.16, 2.05	4.37, 5.63	0.37, 1.07	0.02, 1.00
p value	<0.01	0.03	0.21	<0.01	<0.01	<0.01	0.08	<0.01	<0.01
Participants with genotype data	16.2, 1.79	15.8, 1.20	45.4	100	85.6, 24.1	4.00, 1.98	5.93, 8.94	−0.38, 1.70	0.01, 0.98

Abbreviations: Standard deviation (SD), General Certificate of Secondary Education (GCSE), Diopters (D). Maternal educational levels graded 1–7 (none, primary school, secondary school, vocational certificate/diploma, undergraduate, postgraduate). IQ (Intelligent Quotient) score provided by g (general cognitive ability), which is a composite score of tests of verbal and non-verbal cognitive ability.
